# Supplementary material for: Information-Theoretic Analysis of a Model of CAR-4-1BB-Mediated NFκB Activation
Source: Bull Math Biol. 2023 Dec 1;86(1):5. doi: 10.1007/s11538-023-01232-6 (PMC10691998; doi:10.1007/s11538-023-01232-6)
Supplement: Supplementary file 1 — Supplementary file1 (DOCX 1125 KB) [file 11538_2023_1232_MOESM1_ESM.docx]

**Information-theoretic analysis of a model of CAR-4-1BB-mediated NFκB activation**

Vardges Tserunyan^1^ and Stacey Finley^1,2,3,*^

^1^Department of Quantitative and Computational Biology, University of Southern California, Los Angeles, CA, USA

^2^Alfred E. Mann Department of Biomedical Engineering, University of Southern California, Los Angeles, CA, USA

^3^Mork Family Department of Chemical Engineering and Materials Science, University of Southern California, Los Angeles, CA, USA

^*^Corresponding author: [sfinley@usc.edu](mailto:sfinley@usc.edu)

**SUPPLEMENTARY FIGURES**


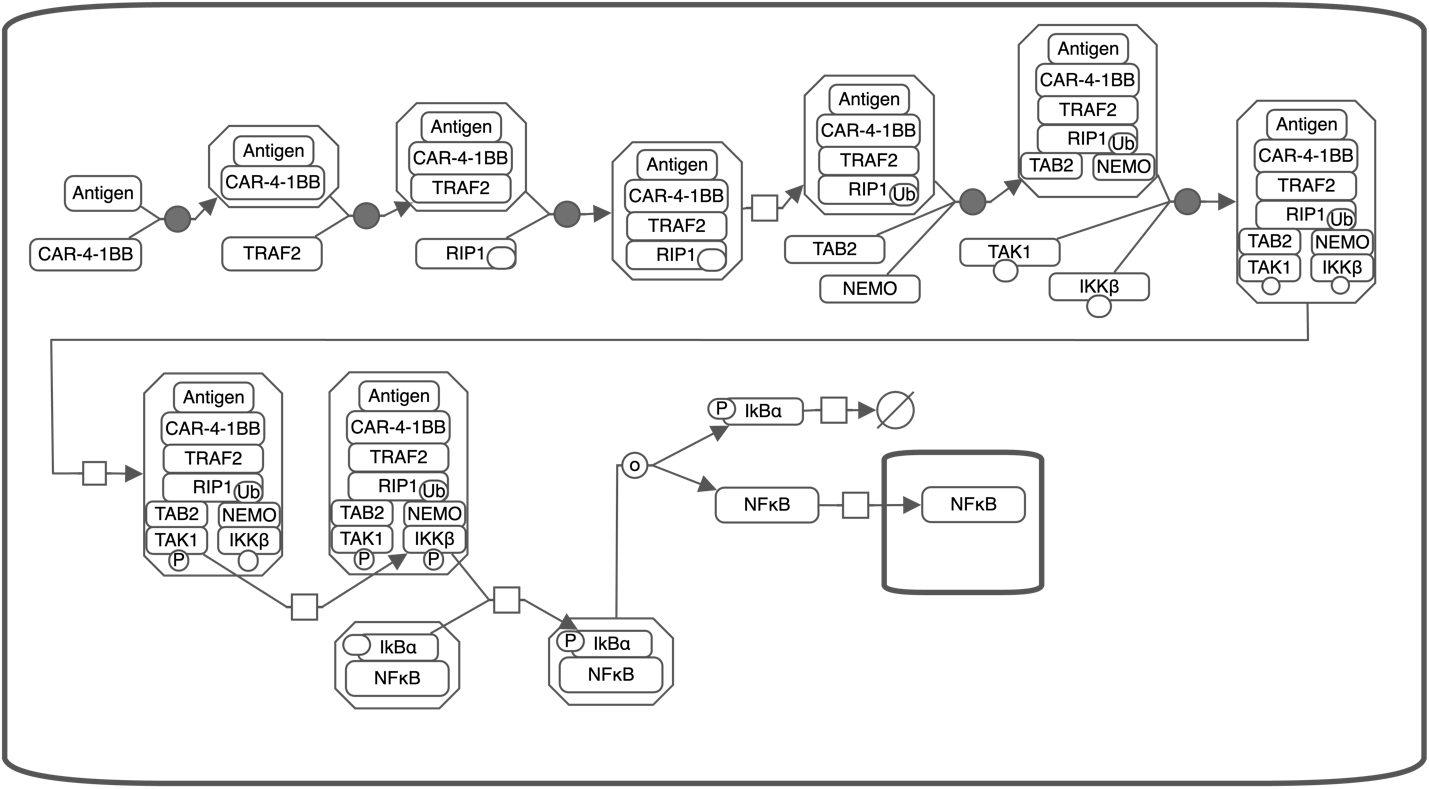


**Membrane/Cytoplasm**

**Nucleus**

**Figure** **S1**: Systems biology graphical notation (SBGN) schematic of the model.


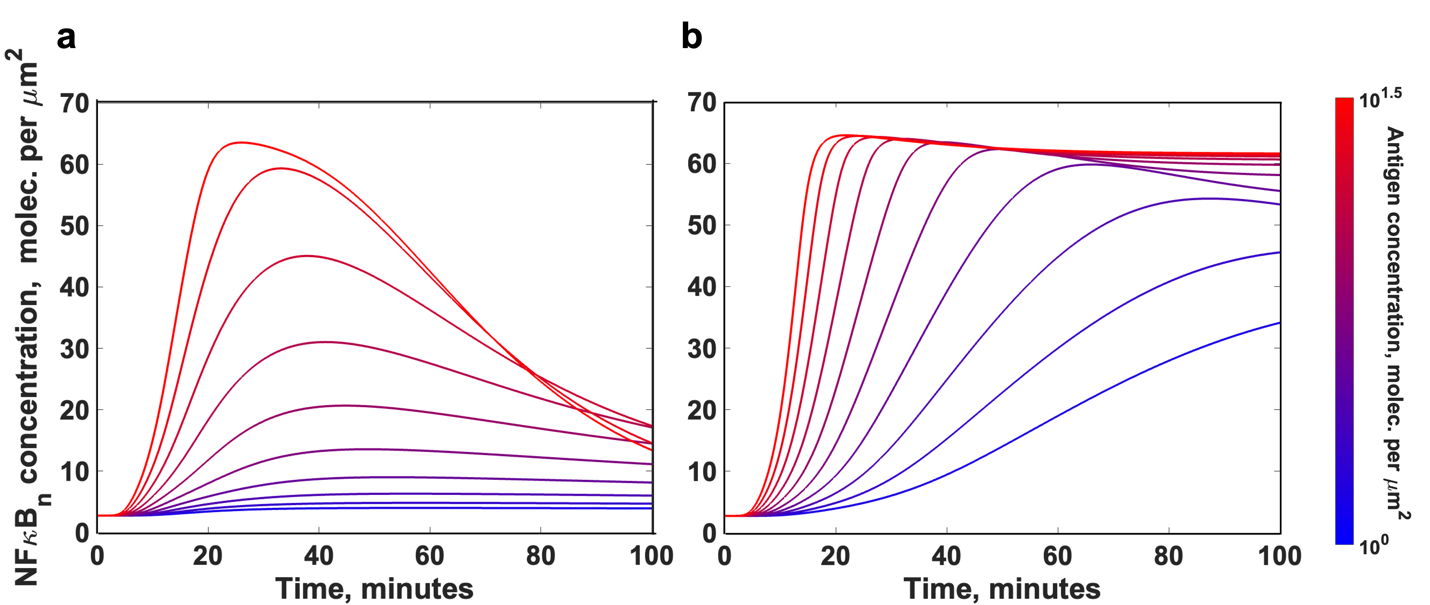


**Figure** **S2**: Time courses for NFκB nuclear abundance induced by different antigen concentrations for the (a) unperturbed model, (b) with disabled IKKβ deactivation.


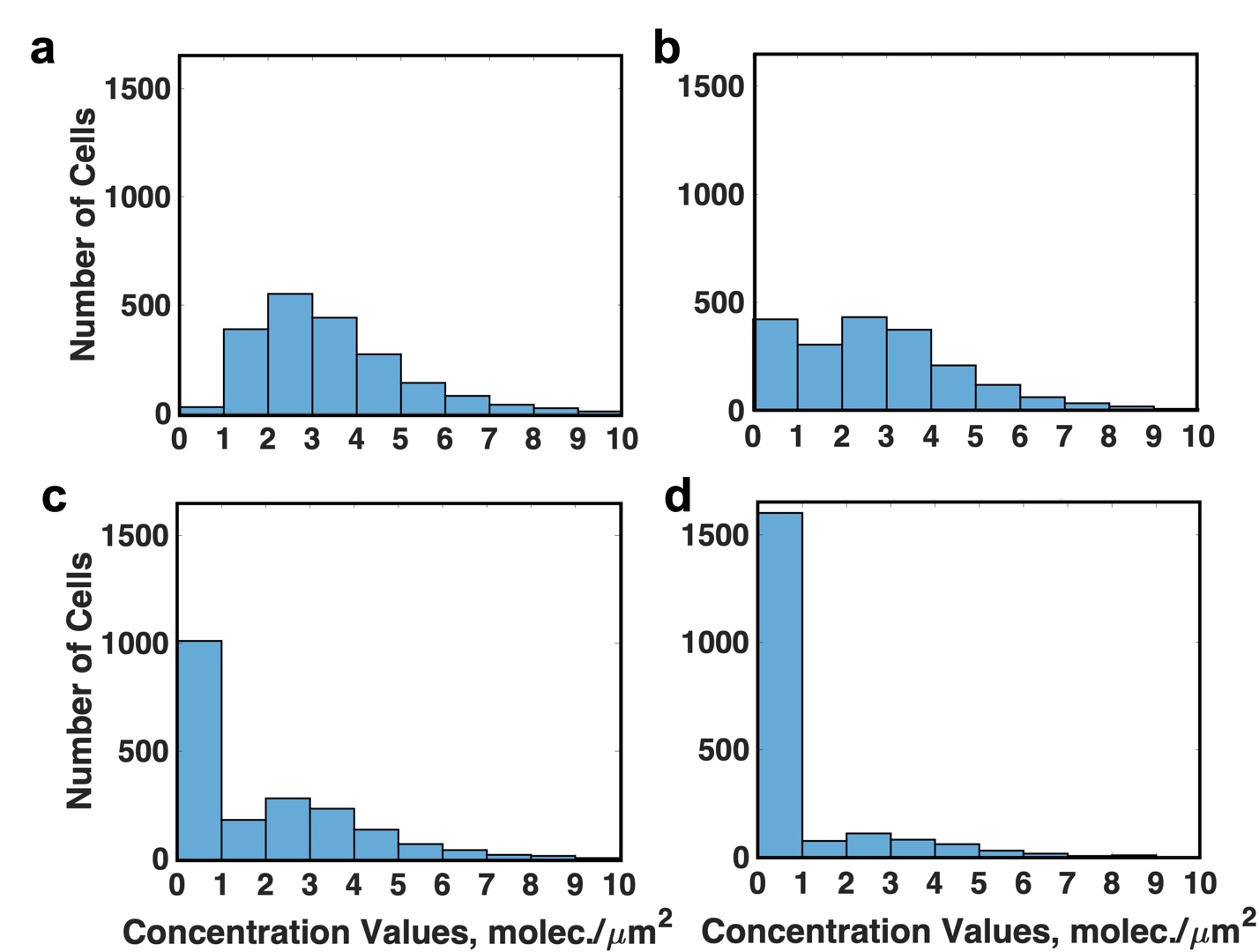


**Figure S3**: Different distributions of antigen concentration for probing the fidelity of CAR-4-1BB-mediated NFκB activation, with identical positive and negative components in varying proportions. (a) 100% cells antigen-positive; (b) 80%; (c) 50%; (d) 20%.


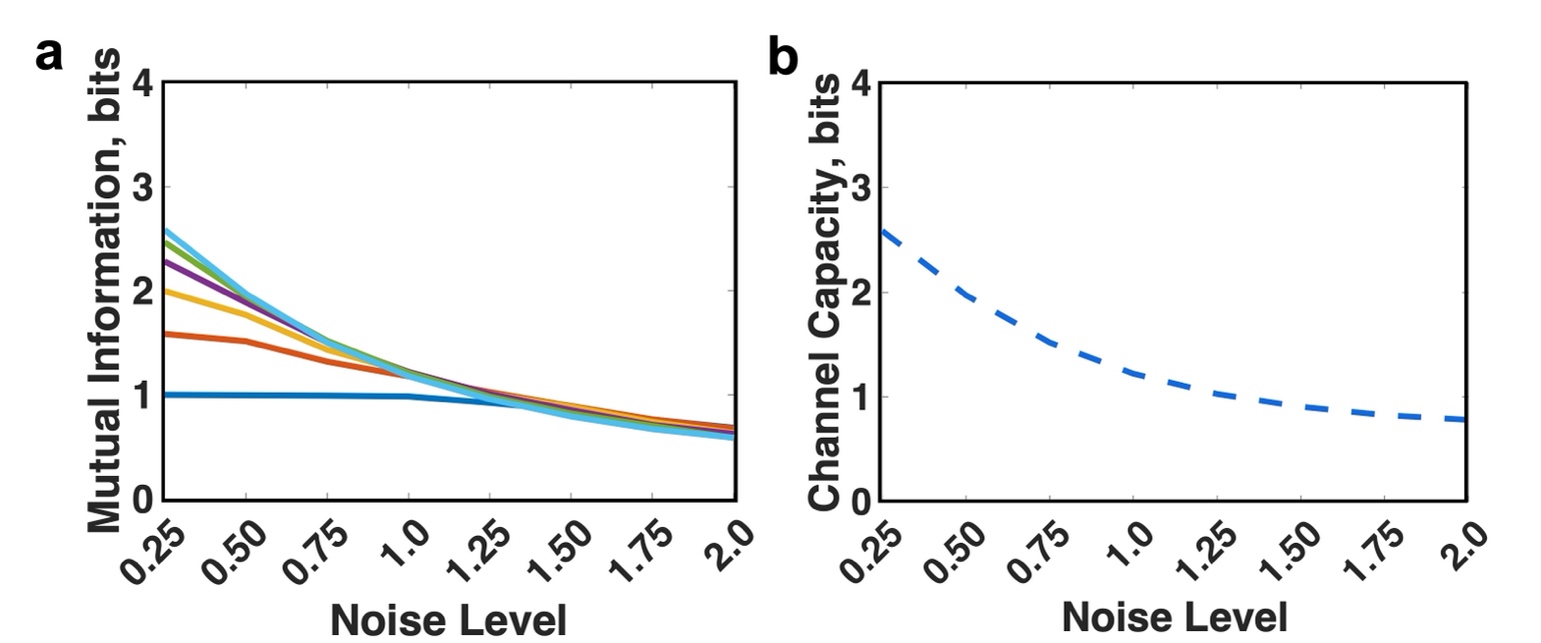


**Figure** **S4**: Example of computing channel capacity. (a) Mutual information between different candidates for a capacity-maximizing antigen distribution and the pathway response (blue, two antigen concentrations; orange, three; yellow, four; purple, five; green, six; cyan, seven). (b) Channel capacity estimated as the maximum mutual information achievable at each noise level, based on A (blue curve identical to that in **Fig. 2a**).


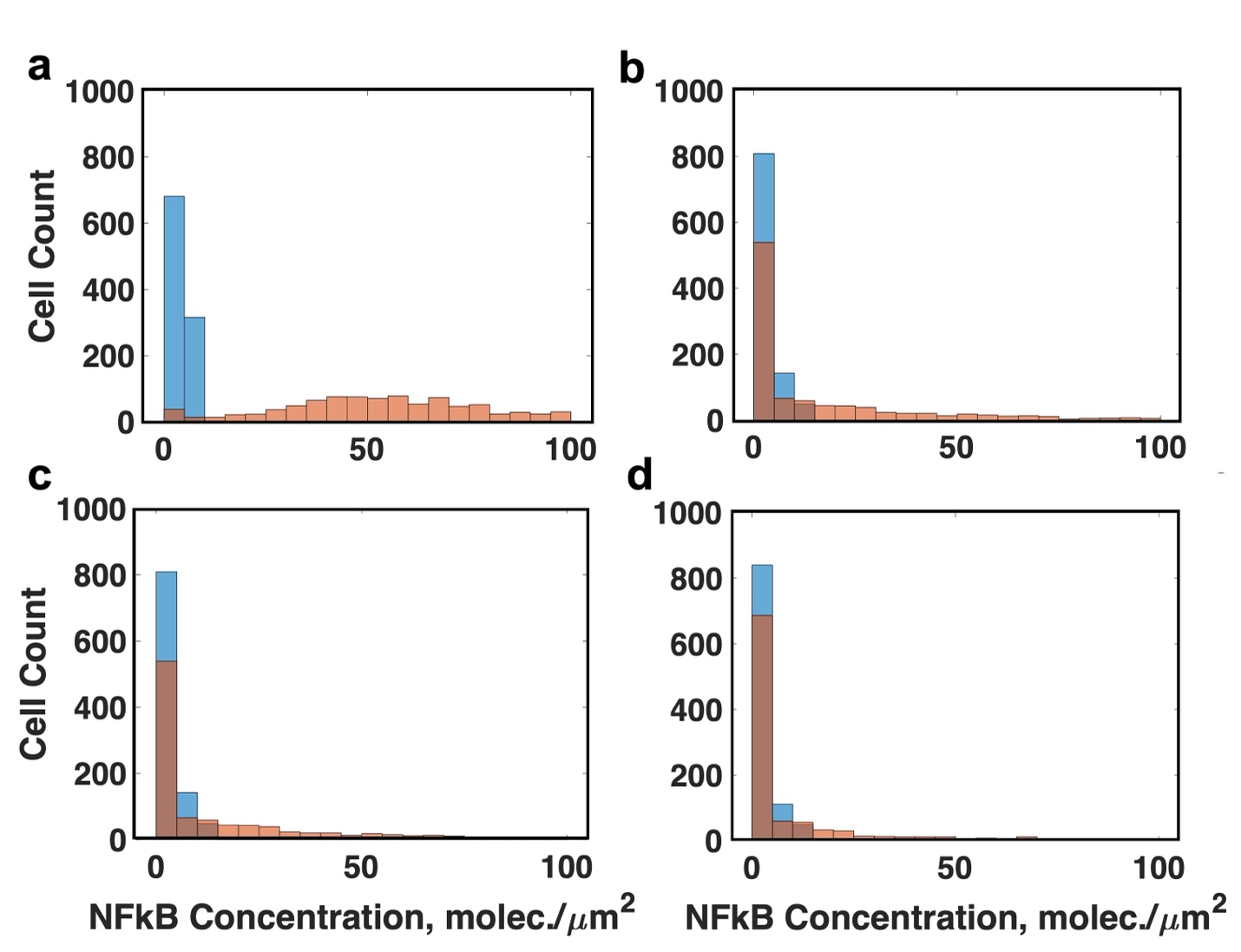


**Figure** **S5**: Deteriorating ability to discern contrasting signals with increasing intrinsic noise. Noise level set at (a) 0.5, (b) 1.0, (c) 1.5, (d) 2.0. Blue shows the unperturbed pathway stimulated with antigen concentration of 0.7 molecules/ µm^2^, orange shows results from stimulation with antigen concentration of 70 molecules/µm^2^. Note that with increasing noise levels, the response distribution of “high” stimulation becomes closer to that of the “low” stimulation. For this reason, most of the response values of “high” stimulation become more characteristic of “low”-stimulated cells.


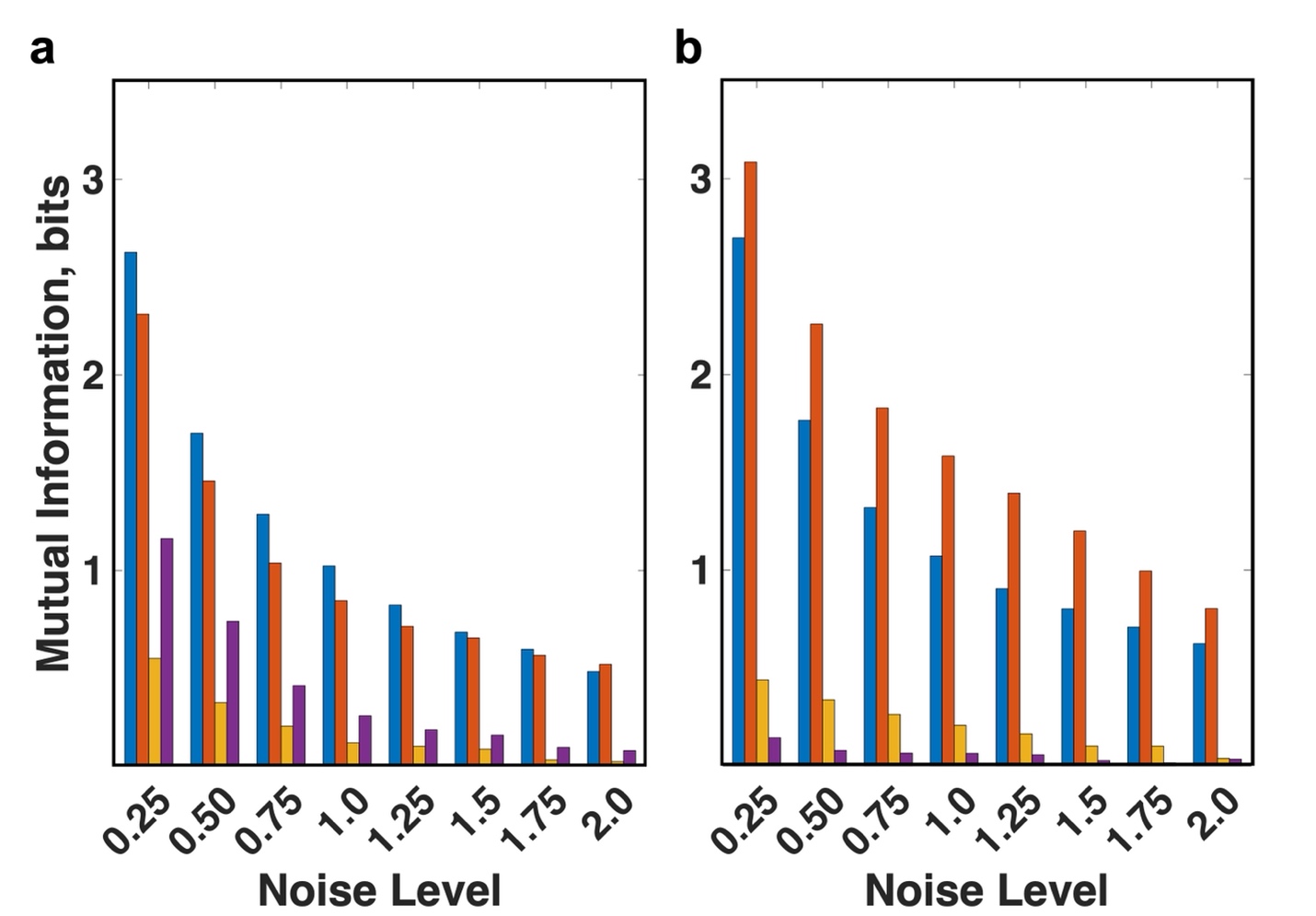


**Figure** **S6**: Mutual information between 100% antigen-positive distribution and various metrics of pathway activation for (a) the unperturbed system and (b) with disabled deactivation of IKKβ. Mutual information between antigen concentration and enzymatically active IKKβ in blue, absolute response of NFκB in orange (if IκBα and NFκB are fixed), absolute response of NFκB in yellow (if IκBα and NFκB are variable), fold change in nuclear NFκB in purple (if IκBα and NFκB are variable).
